# Integument cell gelatinisation—the fate of the integumentary cells in *Hieracium* and *Pilosella* (Asteraceae)

**DOI:** 10.1007/s00709-017-1120-1

**Published:** 2017-05-15

**Authors:** Bartosz J. Płachno, Piotr Świątek, Małgorzata Kozieradzka-Kiszkurno, Zbigniew Szeląg, Piotr Stolarczyk

**Affiliations:** 1 Department of Plant Cytology and Embryology, Jagiellonian University in Kraków, 9 Gronostajowa St., 30-387 Kraków, Poland; 2 Department of Animal Histology and Embryology, University of Silesia in Katowice, 9 Bankowa St., 40-007 Katowice, Poland; 3 Department of Plant Cytology and Embryology, University of Gdańsk, 59 Wita Stwosza St., 80-308 Gdańsk, Poland; 4 Department of Botany, Pedagogical University of Kraków, 3 Podchorążych St., 30-084 Kraków, Poland; 5 Unit of Botany and Plant Physiology, Institute of Plant Biology and Biotechnology, Faculty of Biotechnology and Horticulture, University of Agriculture in Kraków, 29 Listopada 54 Street, 31-425 Kraków, Poland

**Keywords:** Apomixis, Asteraceae, Integument, Lysigenous cavities, Mucilage cells, Ovule, Plasmodesmata, Ultrastructure, Idioblasts

## Abstract

Members of the genera *Hieracium* and *Pilosella* are model plants that are used to study the mechanisms of apomixis. In order to have a proper understanding of apomixis, knowledge about the relationship between the maternal tissue and the gametophyte is needed. In the genus *Pilosella*, previous authors have described the specific process of the “liquefaction” of the integument cells that surround the embryo sac. However, these observations were based on data only at the light microscopy level. The main aim of our paper was to investigate the changes in the integument cells at the ultrastructural level in *Pilosella officinarum* and *Hieracium alpinum*. We found that the integument peri-endothelial zone in both species consisted of mucilage cells. The mucilage was deposited as a thick layer between the plasma membrane and the cell wall. The mucilage pushed the protoplast to the centre of the cell, and cytoplasmic bridges connected the protoplast to the plasmodesmata through the mucilage layers. Moreover, an elongation of the plasmodesmata was observed in the mucilage cells. The protoplasts had an irregular shape and were finally degenerated. After the cell wall breakdown of the mucilage cells, lysigenous cavities that were filled with mucilage were formed.

**Electronic supplementary material:**

The online version of this article (doi:10.1007/s00709-017-1120-1) contains supplementary material, which is available to authorized users.

## Introduction

Members of the genera *Hieracium* L. and *Pilosella* Vaill. are important model plants for understanding the mechanisms of apomixis in angiosperms (e.g. Koltunow et al. 2011a,b; Tucker et al. 2012; Okada et al. 2013; Hand and Koltunow 2014; Hand et al. 2015; Shirasawa et al. 2015; Rabiger et al. 2016; Rotreklová and Krahulcová 2016).

According to Koltunow et al. (1998), the development of the embryo and endosperm in *Hieracium aurantiacum* L. [= *Pilosella aurantiaca* (L.) F. W. Schultz & Sch. Bip.], *H. pilosella* L. [= *P. officinarum* Vaill.] and *P. piloselloides* (Vill.) Soják [= *H. piloselloides* Vill.] coincide with the intensive “liquefaction” of the integument cells that surround the embryo sac. This process was observed in both sexual and apomictic plants and relied on changes in integument cell wall followed by integument cell liquefaction near the endothelium and finally the accumulation of carbohydrate-rich material. According to Koltunow et al. (1998), this material may serve a nutritive role and moreover, they suggested that the accumulation of a large pool of nutrients around the embryo sac might have helped the evolution of the apomictic trait within the genus. This suggestion about a nutritional function of these specific integumentary cells was accepted and repeated by other authors, e.g. Van Baarlen et al. (1999) wrote that the ovules of *Hieracium* and *Taraxacum* contain a protein-rich storage tissue, which nourishes the embryo and reduces the importance of the endosperm function. It was also suggested by these authors that the presence of this tissue might explain the evolution of autonomous embryo development in most of the Asteraceae apomicts. A similar suggestion was repeated in the case of *Taraxacum* and *Chondrilla* by Musiał et al. (2013) and later by Musiał and Kościńska-Pająk (2013). These integumentary cells were called integumentary “nutritive tissue” and its presence and ultrastructure in different members of Asteraceae was discussed by Kolczyk et al. (2014). Although data about the ultrastructure of the integument in both *Hieracium* and *Pilosella* are still lacking, progress has been made in the case of another apomictic genus, *Taraxacum*, which belongs to the same subfamily. Płachno et al. (2016) showed that the “nutritive tissue” (= peri-endothelial tissue) in the *Taraxacum* ovule consists of specialised mucilage cells. During the differentiation of these cells and the deposition of mucilage, the plasmodesmata become elongated and are associated with structures called “cytoplasmic bridges.”

It is well known that the plasmodesmata are plant cell communication channels that are crucial for controlling the intercellular transport of macromolecules such as mRNA, signals including proteins and transcriptional factors (e.g. Oparka 2004; Gursanscky et al. 2011; Hyun et al. 2011). Symplasmic isolation/communication between the ovular sporophytic tissues and the megagametophyte and later the embryo is necessary for successful development (e.g. Ingram 2010; Bencivenga et al. 2011; Marzec and Kurczynska 2008, 2014; Wróbel-Marek et al. 2017 and literature therein). Sporophytic ovule tissues also have an influence on apomixis, e.g. Tucker et al. (2012) showed that in *Pilosella* ovules, sporophytic information is potentiated by the growth of the funiculus and also that polar auxin transport influences ovule development, the initiation of apomixis and the progression of embryo sac. According to Okada et al. (2013), signalling molecules such as the kinases from the sporophytic ovule cells have an influence on the aposporous embryo sac formation in the apomictic *Hieracium* species. Thus, in order to properly understand the symplasmic isolation/communication in *Pilosella* and *Hieracium* ovules, basic knowledge about the ultrastructure of the sporophyte tissues is needed.

It should be stressed that the selection of our research material is not accidental. There are amphimictic diploids and also apomictic polyploids (mitotic diplospory) among the genus *Hieracium*. However, in the genus *Pilosella*, both amphimictic taxa (diploids, sometimes tetra- and hexaploids) and poliploidal facultative apomicts (apospory) are known. Thus, we would like to compare if any differences occur in the integument structure of these genera.

### Aims

The main aim of our paper was to investigate the changes in the integument cells that surround the embryo sac in *Hieracium* and *Pilosella*.

Another question is what happens to the plasmodesmata in these cells. Are the plasmodesmata in the ovule integumentary cells of *Hieracium* and *Pilosella* associated with the cytoplasmic bridges (the thin strands of cytoplasm) like in *Taraxacum* ovules?

We also wanted to investigate whether there is an accumulation of a large pool of nutrients (protein and lipid storage) in the peri-endothelial integument cells that surround the embryo sac.

## Material and methods

### Plant material

**Fig. 1:**
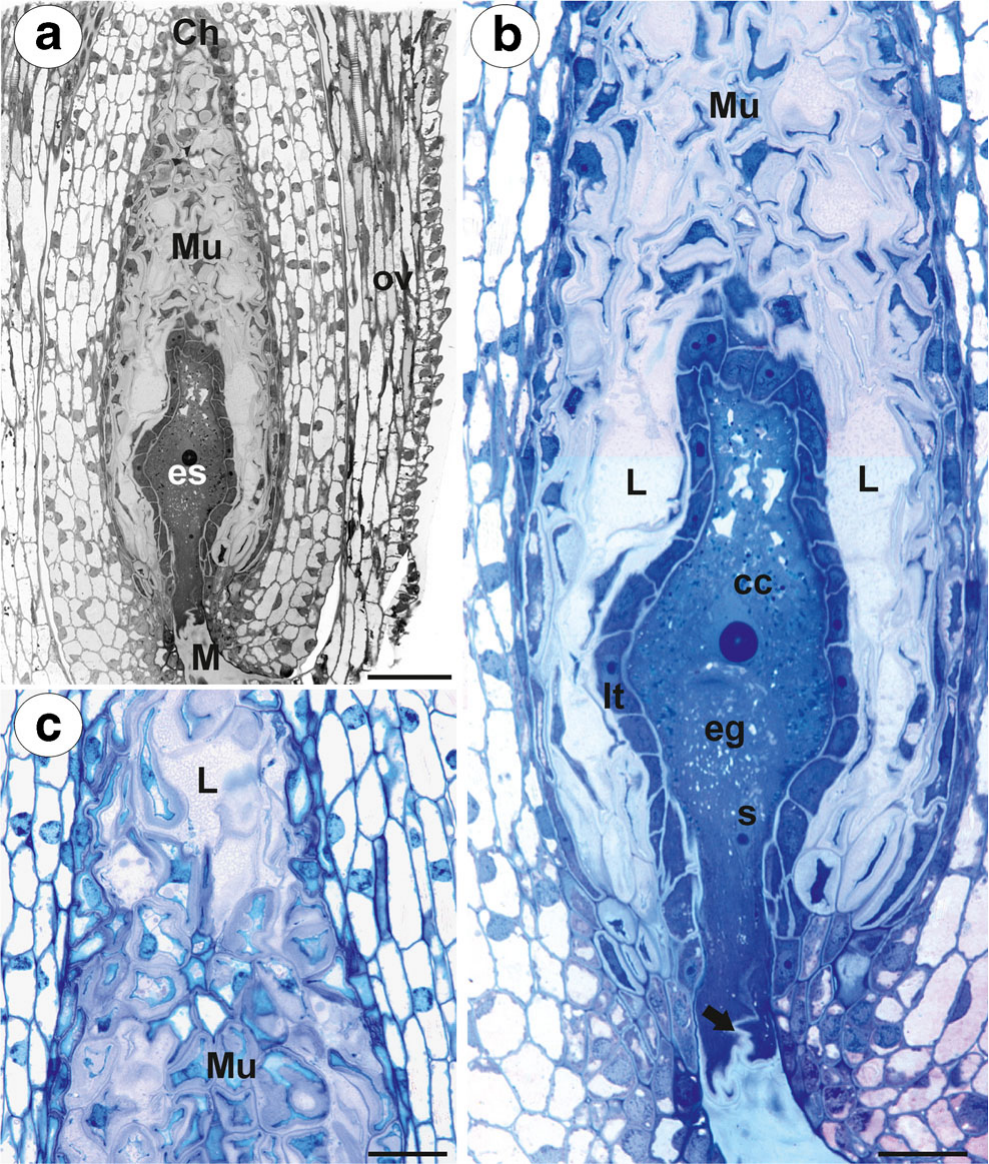
*Pilosella* *officinarum*, Light microscopy. **a** Semithin section through an ovary (*ov*), ovule with an embryo sac (*es*). Peri-endothelial tissue–mucilage cells (*Mu*), micropyle (*M*), chalaza (*Ch*). Bar = 50 μm. **b** Semithin section through an ovule with an embryo sac showing the structure of the periendothelial zone cells (*Mu*) and mucilage cavities (*L*). Egg cell (*eg*), central cell (*cc*), synergids (*s*) synergid filiform apparatus (*arrow*), integumental tapetum (*It*). Bar = 20 μm. **c** Chalazal periendothelial tissue note irregular shape of protoplasts of mucilage cells; mucilage cavities (*L*), bar = 20 μm

**Fig. 2:**
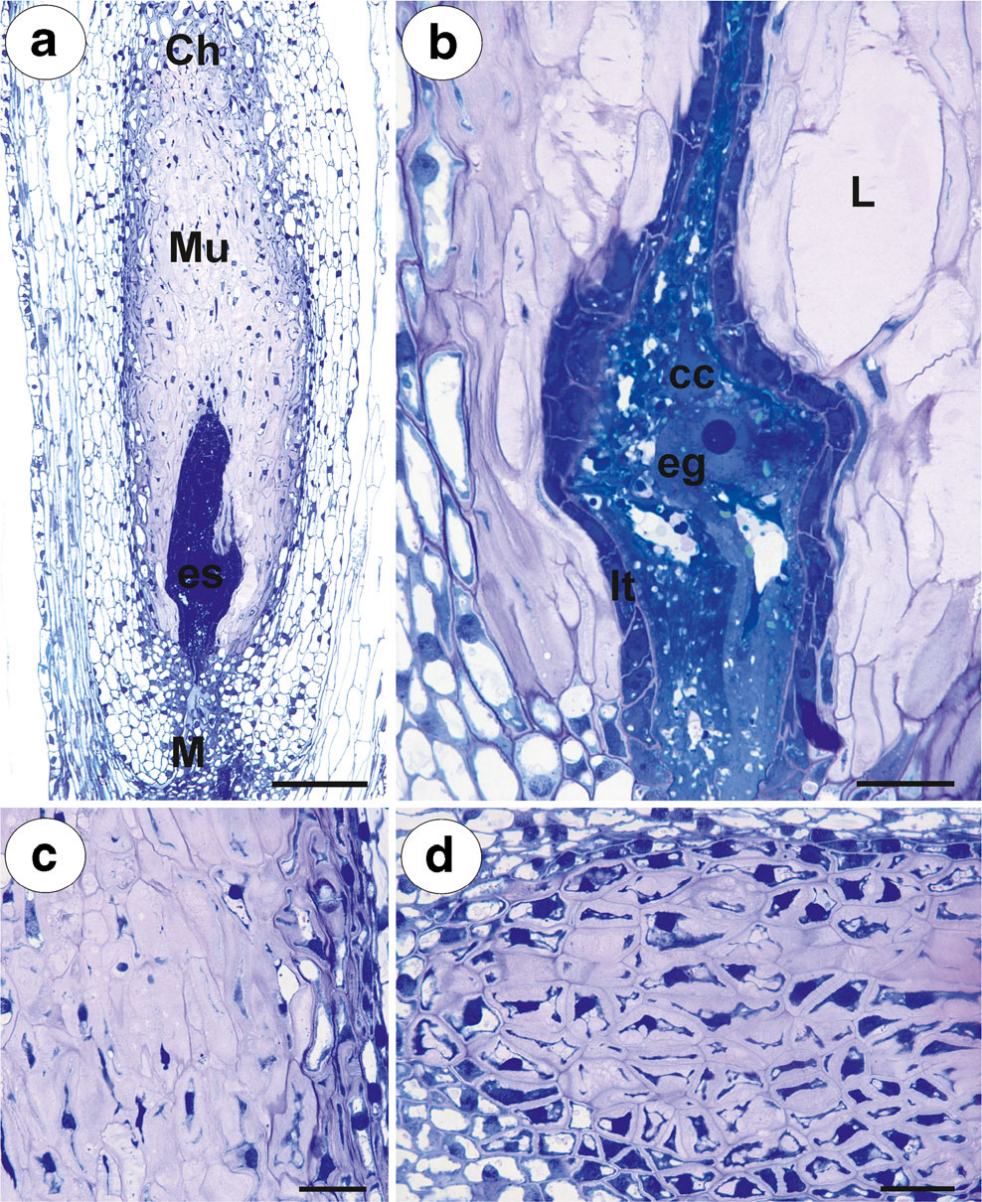
*Hieracium alpinum*, light microscopy. **a** Semithin section through an ovule with an embryo sac showing the structure of the peri-endothelial zone cells–mucilage cells (*Mu*). Embryo sac (*es*), micropyle (*M*), chalaza (*Ch*). Bar = 50 μm. **b** Higher magnification of the embryo sac and periendothelial tissue. Egg cell (eg), central cell (cc), mucilage cavities (L), integumental tapetum (*It*). Bar = 20 μm. **c**, **d** Peri-endothelial mucilage cells in **d** at the chalazal pole. Bar = 20 μm

The plants of an amphimictic *P. officinarum* Vaill. clone for the present study were collected by BJP in their natural habitat in Kokotek near the town of Lubliniec, Poland (tetraploid clone *x* = 9; Sak et al. 2016). Another plant of *P. officinarum* [hexaploid clone *x* = 9, Ilnicki and Szeląg 2011] was collected by ZS in the Mt. Treskovac, Banat, Romania. Plants of *H. alpinum* L. [diploid cytotype *x* = 9, Ilnicki and Szeląg 2011] were collected by ZS in the Retezat Mountains, Southern Carpathians, Romania. In both species, the flowers that were used in this study were harvested before and during anthesis. They contained ovules with mature embryo sacs of the Polygonum type (Figs. 1a, b and 2a, b). Transmission electron microscopy (TEM) analysis was performed on at least three different samples from each species. About 200 TEM pictures were taken and analysed.

### Light and electron microscopy studies

The preparation of the samples for TEM followed the procedure used by Płachno and Świątek (2011) and Kozieradzka-Kiszkurno and Płachno (2012). Semithin sections were stained using aqueous methylene blue with azure II for general histology (Humphrey and Pittman, 1974) for 1–2 min (MB/AII) and examined using an Olympus BX60 microscope. The cytochemical tests included Aniline Blue Black (Jensen, 1962) for proteins and Sudan Black B for lipids (Bronner, 1975). The periodic acid-Schiff (PAS) reaction was used to visualise the total carbohydrates of insoluble polysaccharides (Wędzony 1996).

Ultrathin sections were cut on a Leica Ultracut UCT ultramicrotome. After contrasting with uranyl acetate and lead citrate, the sections were examined using a Hitachi H500 electron microscope at 75 kV in the Faculty of Biology and Environmental Protection, University of Silesia in Katowice and a Jeol JEM 100 SX; JEOL, Tokyo, Japan, at 80 kV in the Department of Cell Biology and Imaging, Institute of Zoology, Jagiellonian University in Kraków.

## Results

**Fig. 3:**
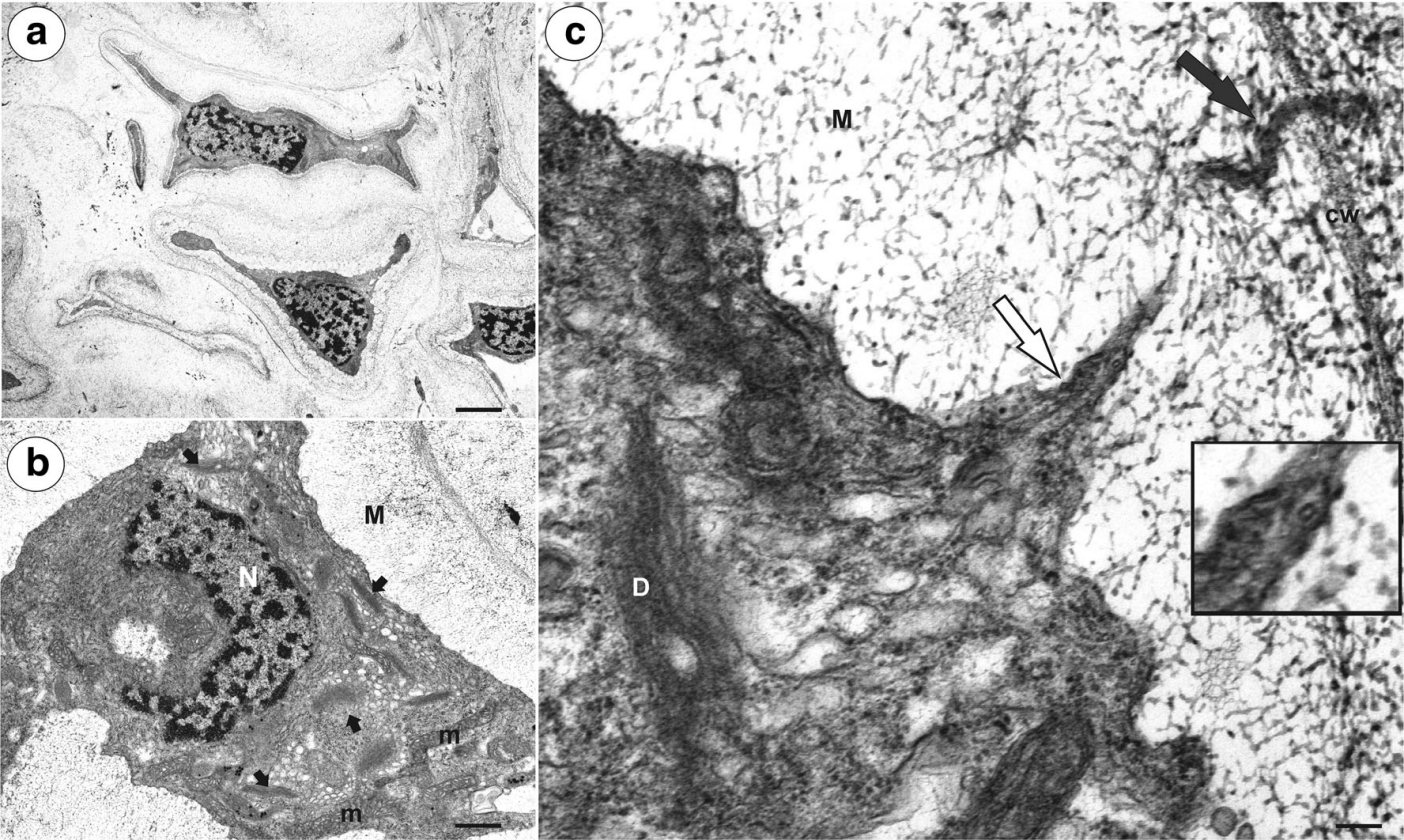
Ultrastructure of the peri-endothelial zone cells in *Pilosella officinarum*, TEM. **a** General ultrastructure of the mucilage cells. Bar = 3.5 μm. **b** Ultrastructure of the mucilage cells: dictyosomes with numerous vesicles (*arrow*), mitochondrion (*m*), nucleus (*N*), mucilage (*M*). Bar = 1 μm. **c** Plasmodesmata connected with the cytoplasmic bridge (*white arrow*); note that the plasmodesma (*black arrow*) is partially embedded in the mucilage (*M*), cell wall (*cw*), dictyosome (*D*). In insert, there is a magnified part of cytoplasmic bridge to show microtubule. Bar = 0.15 μm

**Fig. 4:**
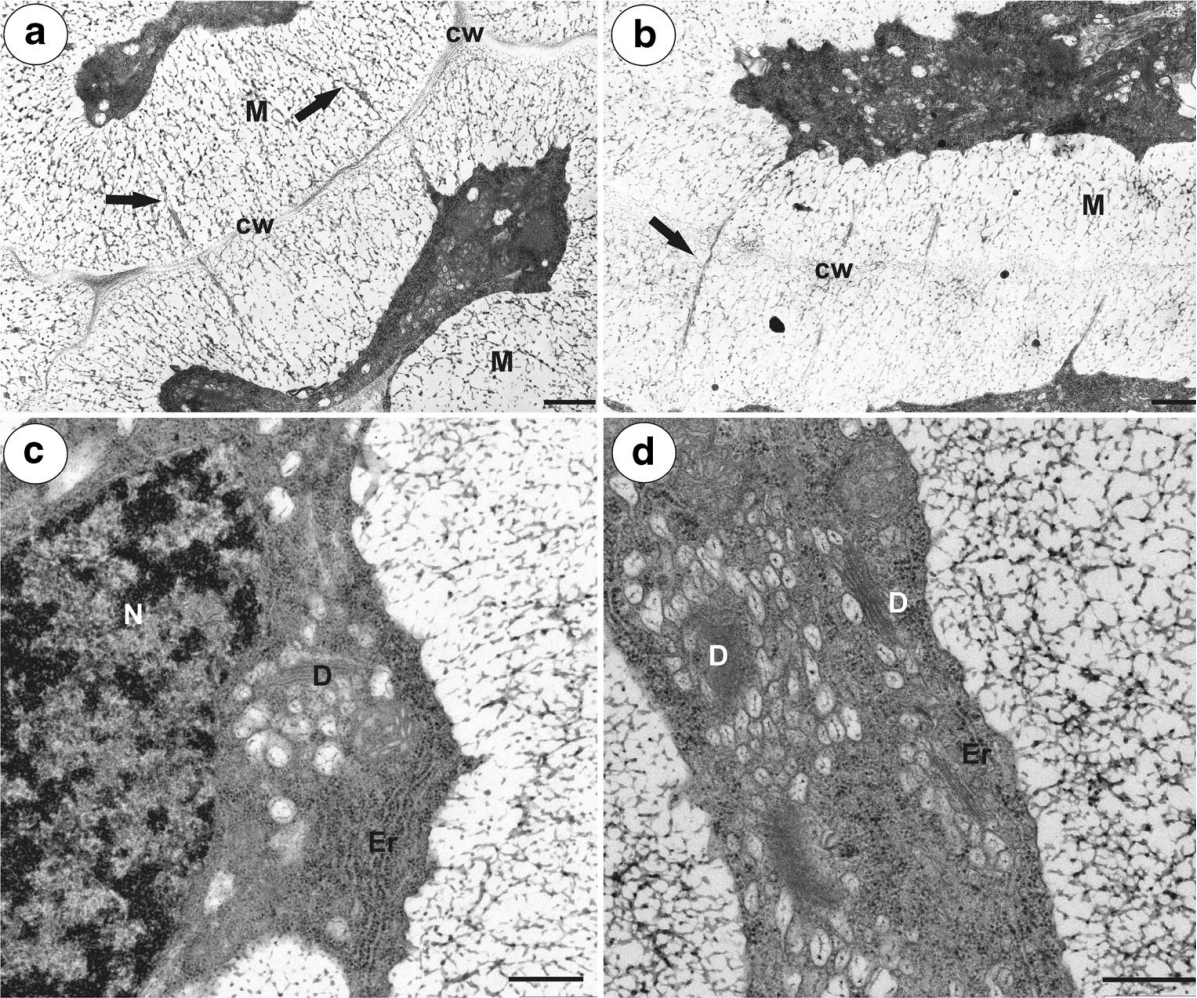
Ultrastructure of the peri-endothelial zone cells in *Hieracium alpinum*, TEM. **a**, **b** General ultrastructure of the mucilage cells. Note cytoplasmic–plasmodesmata connections between the mucilage cells (*black arrows*), mucilage (*M*), cell wall (*cw*). Bar = 0.8 μm and bar = 0.85 μm. **c**, **d** Hypertrophied dictyosomes (*D*) with numerous vesicles containing mucilage. Nucleus (*N*), mitochondria, rough endoplasmic reticulum (*Er*). Both bars = 500 nm

In both species, at the mature female gametophyte stage (Figs. 1a, b and 2a, b), the ovule had a considerably thick, multilayer integument, which had a heterogeneous structure (outer epidermis, highly vacuolated parenchyma, periendothelial tissue, integumentary tapetum; Figs. 1 and 2). The female gametophyte was surrounded by peri-endothelial tissue (Figs. 1 and 2), which was very well developed especially at the chalazal pole of the ovule (Figs. 1a, c and 2a, d). The peri-endothelial tissue consisted of mucilage cells (Figs. 3 and 4). The protoplasts of these cells had an irregular shape (Figs. 3a, b and 4a, b). Mucilage was amorphous, electron translucent with electron-dense reticulate components. It was formed by hypertrophied dictyosomes (Figs. 3b, c and 4c, d) and deposited in the extraplasmatic space between the cell wall and the plasmalemma (Figs. 3b, c and 4a, b). The cytoplasm contained a large nucleus (Fig. 3a, b), many vesicles with mucilage (from the dictyosomes), a rough endoplasmic reticulum and ribosomes (Figs. 3c and 4c, d). Although the mucilage pushed the protoplast to the centre of the cell, the mucilage cells were still symplasmically connected (Figs. 3c and 4a, b).

**Fig. 5:**
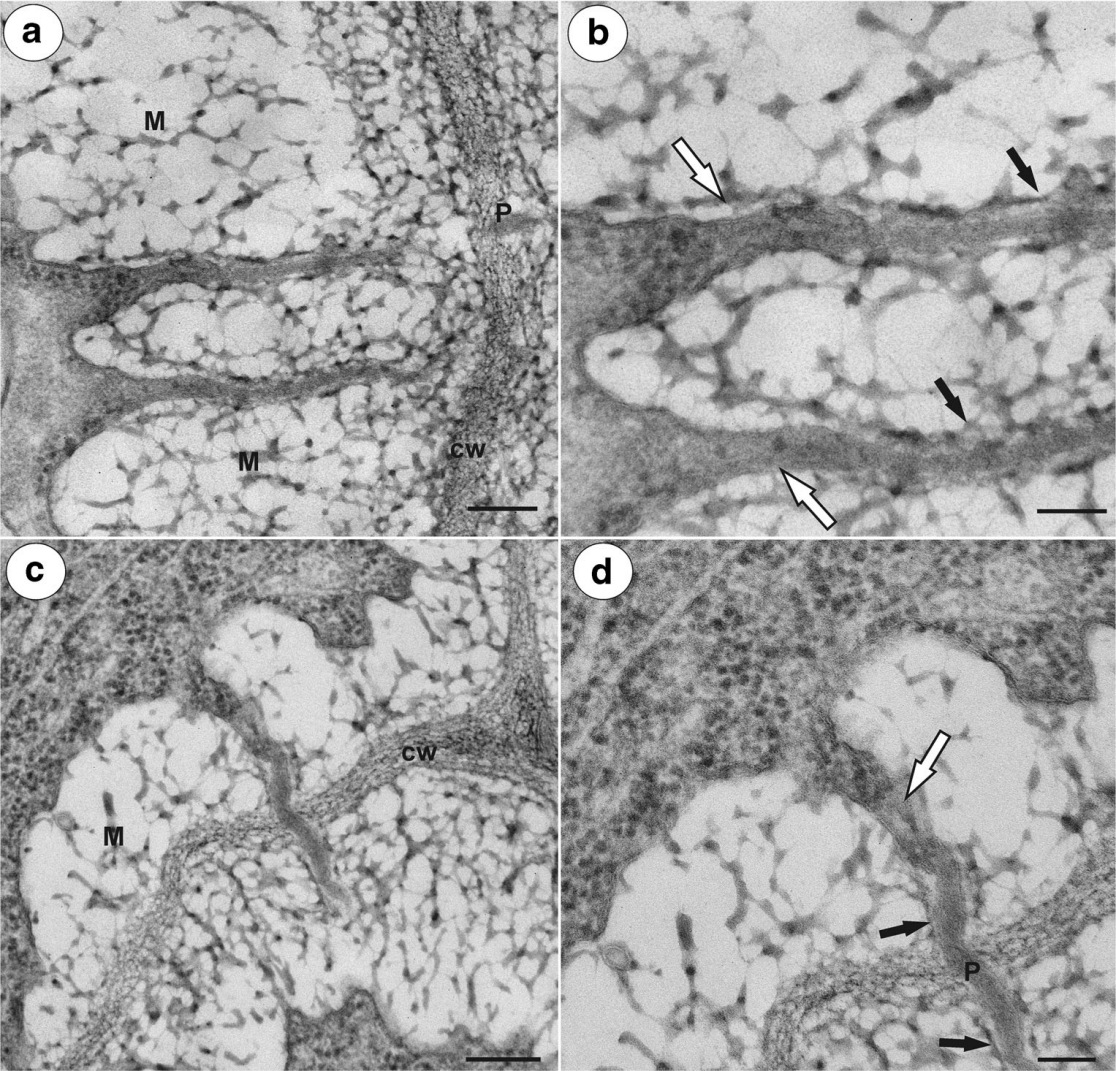
Cytoplasmic connections in the integument mucilage cells of *Hieracium alpinum*, TEM. **a**–**d** Plasmodesmata connected with the cytoplasmic bridges (*white arrow*). Note that the plasmodesmata (*black arrow*) are partially embedded in the mucilage (*M*), “typical” plasmodesmata in cell wall (*P*). In the cytoplasmic bridge, ribosomes are visible. **a**, **c** Bar = 200 nm. **b**, **d** Bar = 100 nm

**Fig. 6:**
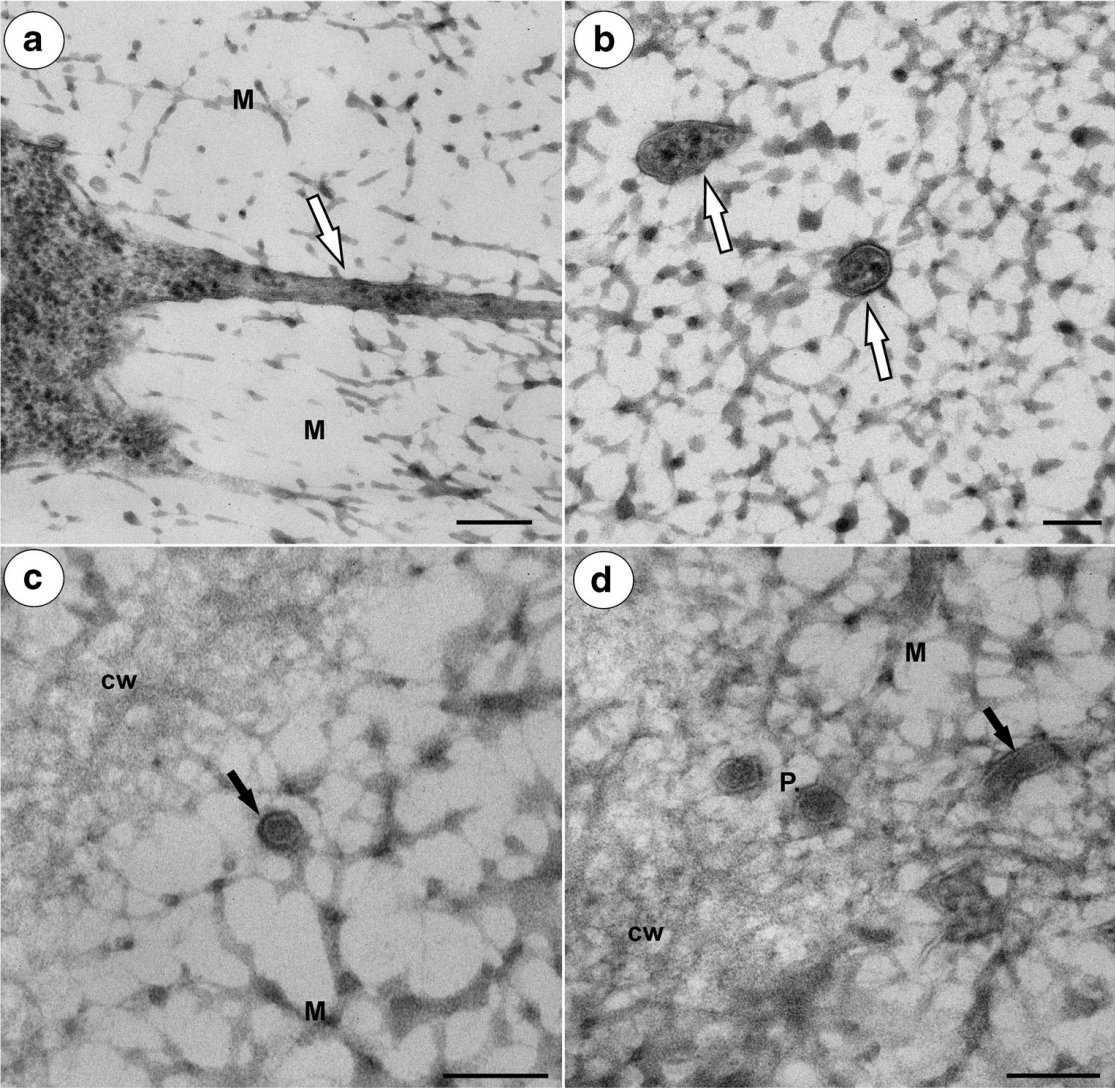
Cytoplasmic connections in the integument mucilage cells of *Hieracium alpinum*, TEM. **a** Longitudinal section through a cytoplasmic bridge (*white arrow*) embedded in the mucilage (*M*). Bar = 200 nm. **b** Near transverse section through the cytoplasmic bridges (*white arrows*) embedded in the mucilage. Bar = 100 nm. **c** Transverse section through the plasmodesma (*black arrow*) in the mucilage (*M*) close to the cell wall (*cw*). **d** Transverse section through the plasmodesmata (*P*) in the cell wall (*cw*) and a longitudinal section through the plasmodesmata (*black arrow*) in the mucilage (*M*). Bar = 100 nm

**Fig. 7:**
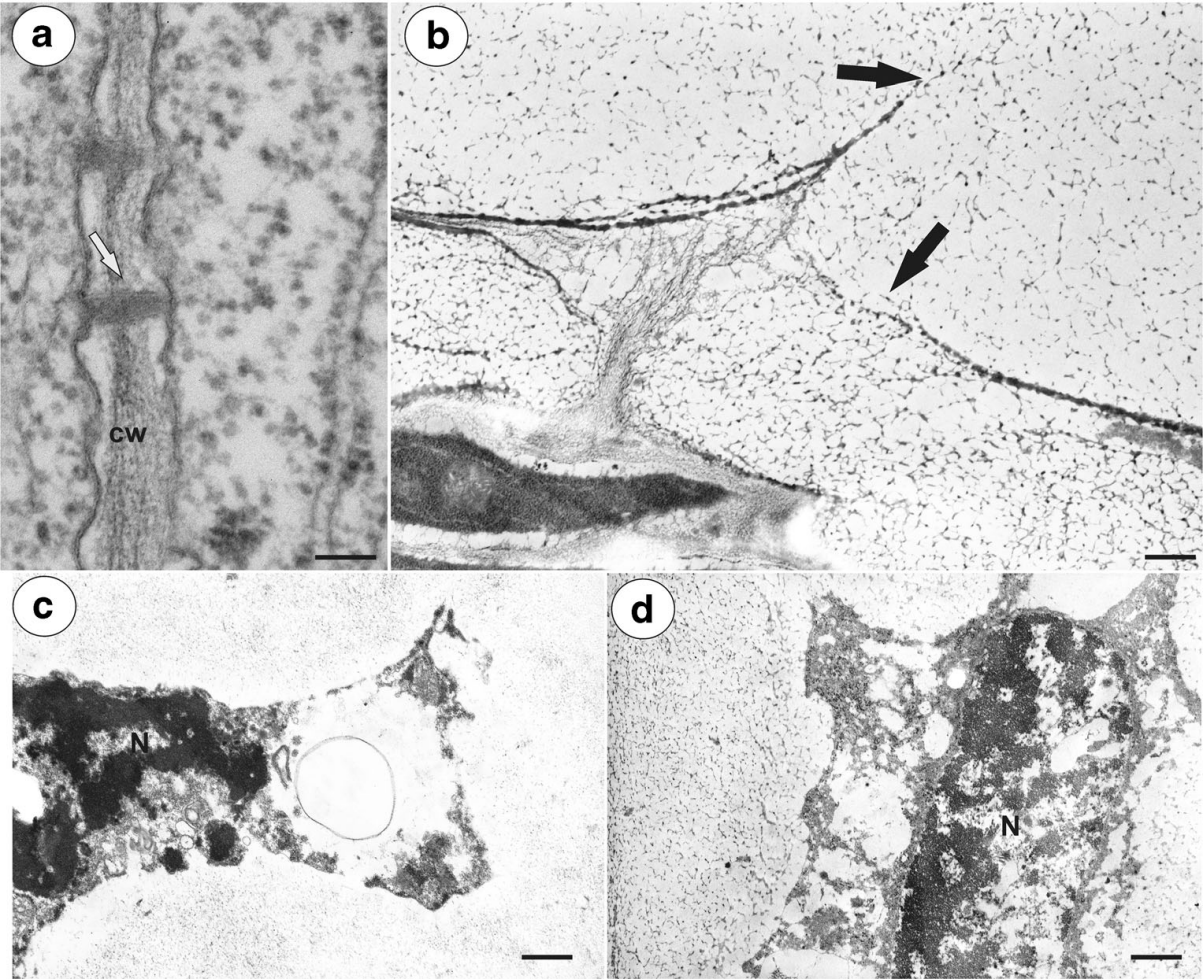
Ultrastructure of the integument cells, TEM. **a** *Hieracium alpinum.* Longitudinal section through the plasmodesmata (*white arrow*) of the non-mucilage cells. Cell wall (*cw*). Bar = 100 nm. **b** *Hieracium alpinum.* Breakdown of the cell wall (*black arrows*) between adjacent mucilage cells. Bar = 0.4 μm. **c** *Pilosella officinarum.* Degradation of the mucilage cell protoplast, nucleus (*N*). Bar = 0.7 μm. **d** *Hieracium alpinum.* Degradation of the mucilage cell protoplast, nucleus (*N*). Bar = 0.9 μm

In both species, there were thin strands of cytoplasm (cytoplasmic bridges) that connected the protoplast with the plasmodesmata (Figs. 3c, 5a–d, and 6a). In the cytoplasmic bridges, microtubules and ribosomes were visible (Figs. 3c, 5b–d, and 6a, b). In the mucilage close to the primary wall, cytoplasmic bridges were connected with the plasmodesmata, which had passed the plasmodesmata in the primary cell wall (Fig. 5a–d). On the transverse and longitudinal sections of the plasmodesmata, the plasmalemma and the desmotubule were clearly visible in the mucilage (Fig. 6c, d). The structure of these plasmodesmata was similar to the typical plasmodesmata in the cell walls of non-mucilage parenchyma cells (Fig. 7a).

A breakdown of the cell wall between the adjacent mucilage cells occurred (Fig. 7b), after which lysigenous cavities that were filled with mucilage were formed (Figs. 1b, c and 2b). The protoplast of mucilage cells was finally degraded (Fig. 7c, d).

The cytochemical tests for the storage lipids and protein gave negative results in the case of the mucilage cells (not shown). However, we observed small lipid droplets in the cytoplasm of the mucilage cells using TEM (Supplementary material 1). There was positive staining after periodic-acid-Schiff reaction. The total carbohydrates of insoluble polysaccharides (including mucilage carbohydrates) stain pink to purplish red (Supplementary material 2).

## Discussion

We showed that the intensive “liquefaction” of the integument cells surrounding the embryo sac, which was previously described by Koltunow et al. (1998), was in the fact gelatinisation: an accumulation of the mucilage in the cells and later the formation of lysigenous cavities that were filled with mucilage. Koltunow et al. (1998) wrote that the material that was accumulated during the intensive changes of the integument cells surrounding the embryo sac was carbohydrate-rich (positive staining after periodic-acid-Schiff reaction). This also agreed with our observation that this material is mucilage. The ultrastructure of the mucilage in *Hieracium* and *Pilosella* is similar to the previously observed mucilage in the integument mucilage cells of other Asteraceae genera—*Taraxacum*, *Onopordum*, *Solidago*, *Chondrilla* and *Bellis* (Płachno et al. 2016; Kolczyk et al. 2014, 2016 and literature therein). However, our results disagree with Van Baarlen et al. (1999) who wrote that the ovules of *Hieracium* contain a protein-rich storage tissue, because we did not find protein bodies or protein storage vacuoles in the periendothelial tissue. However, during the gelatinisation of the periendothelial cells and the degeneration of their protoplasts, some nutrients might be released and transported to the female gametophyte. But, such a hypothesis requires experimental confirmation. Moreover, the mucilage in the ovules and seeds may have a different function and be storage for water like the mucilage in cacti cells (Nobel et al. 1992).

Although the peri-endothelial cell ultrastructure and mucilage deposition that were results obtained in this study resemble those in *Taraxacum* (Płachno et al. 2016), there are some differences. In both *Hieracium* and *Pilosella*, the formation of lysigenous cavities occurs at the mature female gametophyte stage, while in *Taraxacum*, the peri-endothelial cells still retain individuality at the mature female gametophyte stage (see Fig. 1a in Płachno et al. 2016) and the formation of these cavities occurs later, during embryogenesis (see Fig. 1c, din Gawecki et al. 2017). Cooper and Brink (1949) also described the disintegration of the peri-endothelial cells in *Taraxacum* during embryogenesis.

The breakdown of the cell wall between the mucilage cells that was observed here has also been described in other plants that are not related to the Asteraceae, e.g. *Hibiscus schizopetalus* (Malvaceae) (Bakker and Gerritsen 1992) and *Cinnamomum* (Lauraceae) (Bakker et al. 1991). However, in *H. schizopetalus*, the local breakdown of the cell wall between the mucilage and neighbouring non-mucilage cells has also been observed many times (Bakker and Gerritsen 1992). In *Cinnamomum* mucilage cells, the local breakdown of the cell wall is not common due to the occurrence of a suberised cell wall layer (Bakker et al. 1991). A suberised wall layer does not occur in the mucilage cells of *Hibiscus* (Bakker and Gerritsen 1992), *Taraxacum* (Płachno et al. 2016) or in *Hieracium* and *Pilosella*.

### Plasmodesmata in mucilage idioblasts

Unfortunately, there are only a few studies about the plasmodesmata in mucilage idioblasts. In the mucilage cells of *C. verum* and *Annona muricata* (Annonaceae), the plasmodesmata show a bulge on the idioblast side of the cell wall. These plasmodesmata become occluded by the mucilage, and according to Bakker and Baas (1993), symplasmic transport is presumably blocked. However, in the mucilage cells of *C. burmanni*, Bakker et al. (1991) noted that in a few cases, connections between the plasmodesma and the cytoplasmic strand that was embedded in the mucilage occurred. Our observation that the plasmodesmata in the mucilage cells are linked to the protoplast via cytoplasmic bridges in *Hieracium* and *Pilosella* are in agreement with a similar situation that was observed in *Taraxacum* mucilage cells (Płachno et al. 2016). Like in *Taraxacum*, our ultrastructural documentation here indicates that there was an elongation of the primary plasmodesmata that correlated with an increase in the thickness of the mucilage. The concept that the plasmodesmata may undergo elongation is not new and was proposed by Ehlers and Kollmann (1996). Glockmann and Kollmann (1996) also documented an elongation of the primary plasmodesmata that correlated with an increase in the thickness of the wall in the Strasburger cells of the needles of *Metasequoia*.

In order to maintain the intercellular communication between integument cells in *Hieracium* and *Pilosella* during mucilage deposition, the primary plasmodesmata have to be elongated. We propose here a model of elongation of primary plasmodesmata in the mucilage idioblasts: a part of cytoplasmic strand which is embedded in mucilage and has contact with the primary plasmodesma become increasingly constricted and develop into plasmodesmal strand. The enclosed ER cisternae inside this strand, which is connected with desmotubule, is transformed into the plasmodesmal desmotubule. This process is similar to elongation of primary plasmodesmata during the thickening growth of the cell walls proposed by Ehlers and Kollmann (1996, 2001); however, with one major difference, that in the mucilage idioblasts, cytoplasmic strands enclosing ER cisternae are in mucilage.

## Conclusions

- We showed that the liquefaction process of the integument cells surrounding the embryo sac in *Hieracium* and *Pilosella* was in the fact of gelatinisation: an accumulation of mucilage in the cells and later the formation of lysigenous cavities that are filled with mucilage.
- The plasmodesmata in the mucilage cells are linked to the protoplast via cytoplasmic bridges, which suggests that they are functional. Our observation may indicate that there is an elongation of the primary plasmodesmata that is correlated with the mucilage deposition.
- Because the mucilage cells of *Hieracium* and *Pilosella* lack storage proteins, the mucilage may perform the role of water storage or may be a source of carbohydrates for the gametophyte and embryo.

## Electronic supplementary material

Supplementary material A. *Hieracium alpinum*, TEM. Ultrastructure of the mucilage cells: lipid droplet (Lp), mucilage (M), endoplasmic reticulum (Er); bar = 200 nm. B *Pilosella officinarum*, Light microscopy. Ovule after PAS reaction, note PAS-positive material in mucilage cells (M); embryo sac (es), mucilage cavities (L); bar = 10 μm. (GIF 142 kb)

High resolution image (TIFF 17239 kb)

## References

[CR1] Bakker ME, Baas P (1993). Cell walls in oil and mucilage cells. Acta Bot Neerl.

[CR2] Bakker ME, Gerritsen AF (1992). The development of mucilage cells in *Hibiscus schizopetalus*. Acta Bot Neerl.

[CR3] Bakker ME, Gerritsen AF, van der Schaaf PJ (1991). Development of oil and mucilage cells in *Cinnamomum burmanni*. An ultrastructural study. Acta Bot Neerl.

[CR4] Bencivenga S, Colombo L, Masiero S (2011). Cross talk between the sporophyte and the megagametophyte during ovule development. Sex Plant Reprod.

[CR5] Bronner R (1975). Simultaneous demonstration of lipid and starch in plant tissues. Stain Technol.

[CR6] Cooper DC, Brink RA (1949). The endosperm-embryo relationship in the autonomous apomict, *Taraxacum officinale*. Bot Gaz.

[CR7] Ehlers K, Kollmann R (1996). Formation of branched plasmodesmata in regenerating *Solanum nigrum—*protoplasts. Planta.

[CR8] Ehlers K, Kollmann R (2001). Primary and secondary plasmodesmata: structure, origin, and functioning. Protoplasma.

[CR9] Gawecki R, Sala K, Kurczyńska EU, Świątek P, Płachno BJ (2017) Immunodetection of some pectic, arabinogalactan proteins and hemicelluloses epitopes in the micropylar transmitting tissue of apomictic dandelions (Taraxacum, Asteraceae, Lactuceae). Protoplasma 254:657–668. doi:10.1007/s00709-016-0980-0 10.1007/s00709-016-0980-0 PMC5309284 27154759

[CR10] Glockmann C, Kollmann R (1996). Structure and development of cell connections in the phloem of *Metasequoia glyptostroboides* needles. I. Ultrastructural aspects of modified primary plasmodesmata in Strasburger cells. Protoplasma.

[CR11] Gursanscky NR, Searle IR, Carroll BJ (2011). Mobile microRNAs hit the target. Traffic.

[CR12] Hand ML, Koltunow AM (2014). The genetic control of apomixis: asexual seed formation. Genetics.

[CR13] Hand ML, Vít P, Krahulcová A, Johnson SD, Oelkers K, Siddons H (2015). Evolution of apomixis loci in *Pilosella* and *Hieracium* (Asteraceae) inferred from the conservation of apomixis-linked markers in natural and experimental populations. Hered.

[CR14] Humphrey CD, Pittman FE (1974). A simple methylene blue-azure II basic fuchsin stain for epoxy-embedded tissue sections. Stain Technol.

[CR15] Hyun TK, Uddin MN, Rim Y, Kim J-Y (2011). Cell-to-cell trafficking of RNA and RNA silencing through plasmodesmata. Protoplasma.

[CR16] Ilnicki T, Szeląg Z (2011). Chromosome numbers in *Hieracium* and *Pilosella* (Asteraceae) from central and southeastern Europe. Acta Biol Cracov Ser Bot.

[CR17] Ingram GC (2010). Family life at close quarters: communication and constraint in angiosperm seed development. Protoplasma.

[CR18] Jensen WA (1962). Botanical histochemistry.

[CR19] Kolczyk J, Stolarczyk P, Płachno BJ (2014). Comparative anatomy of ovules in *Galinsoga*, *Solidago* and *Ratibida* (Asteraceae). Acta Biol Cracov Ser Bot.

[CR20] Kolczyk J, Stolarczyk P, Płachno BJ (2016). Ovule structure of scotch thistle *Onopordum acanthium* L. (Cynareae, Asteraceae). Acta Biol Cracov Ser.

[CR21] Koltunow AM, Johnson SD, Bicknell RA (1998). Sexual and apomictic development in *Hieracium*. Sex Plant Reprod.

[CR22] Koltunow AM, Johnson SD, Okada T (2011). Apomixis in hawkweed: Mendel’s experimental nemesis. J Exp Bot.

[CR23] Koltunow AM, Johnson SD, Rodrigues JC, Okada T, Hu Y, Tsuchiya T (2011). Sexual reproduction is the default mode in apomictic *Hieracium* subgenus *Pilosella*, in which two dominant loci function to enable apomixis. Plant J.

[CR24] Kozieradzka-Kiszkurno M, Płachno BJ (2012). Are there symplastic connections between the endosperm and embryo in some angiosperms?—A lesson from the Crassulaceae family. Protoplasma.

[CR25] Marzec M, Kurczynska EU (2008). Symplasmic communication/isolation and plant cell differentiation (in Polish). Postepy Biol Komorki.

[CR26] Marzec M, Kurczynska E (2014). Importance of symplasmic communication in cell differentiation. Plant Sig & Beh.

[CR27] Musiał K, Kościńska-Pająk M (2013). Ovules anatomy of selected apomictic taxa from Asteraceae family. Mod Phytomorphol.

[CR28] Musiał K, Płachno BJ, Świątek P, Marciniuk J (2013). Anatomy of ovary and ovule in dandelions (*Taraxacum*, Asteraceae). Protoplasma.

[CR29] Nobel PS, Cavelier J, Andrade JL (1992). Mucilage in cacti: its apoplastic capacitance, associated solutes and influence on tissue water relations. J Exp Bot.

[CR30] Okada T, Hu Y, Tucker MR, Taylor JM, Johnson SD, Spriggs A (2013). Enlarging cells initiating apomixis in *Hieracium praealtum* transition to an embryo sac program prior to entering mitosis. Plant Physiol.

[CR31] Oparka KJ (2004). Getting the message across: how do plant cells exchange macromolecular complexes?. Trends Plant Sci.

[CR32] Płachno BJ, Świątek P (2011). Syncytia in plants: cell fusion in endosperm-placental syncytium formation in *Utricularia* (Lentibulariaceae). Protoplasma.

[CR33] Płachno BJ, Kurczyńska E, Świątek P (2016). Integument cell differentiation in dandelions (*Taraxacum*, Asteraceae, Lactuceae) with special attention paid to plasmodesmata. Protoplasma.

[CR34] Rabiger DS, Taylor JM, Spriggs A (2016). Generation of an integrated *Hieracium* genomic and transcriptomic resource enables exploration of small RNA pathways during apomixis initiation. BMC Biol.

[CR35] Rotreklová O, Krahulcová A (2016). Estimating paternal efficiency in an agamic polyploid complex: pollen stainability and variation in pollen size related to reproduction mode, ploidy level and hybridogenous origin in *Pilosella* (Asteraceae). Folia Geobot.

[CR36] Sak D, Janas A, Musiał K, Płachno BJ (2016). Sexual reproductive traits in tetraploid *Pilosella officinarum* (Asteraceae, Cichorioideae): DIC microscope study of cleared whole-mount tissue. XXXII Conference on Embryology Plants Animals Humans, May 18–21, 2016, Wojsławice, Poland. Acta Biol Cracov Ser Bot.

[CR37] Shirasawa K, Hand ML, Henderson ST, Okada T, Johnson SD, Taylor JM (2015). A reference genetic linkage map of apomictic *Hieracium* species based on expressed markers derived from developing ovule transcripts. Ann Bot.

[CR38] Tucker MR, Okada T, Johnson SD, Takaiwa F, Koltunov AMG (2012). Sporophytic ovule tissues modulate the initiation and progression of apomixis in *Hieracium*. J Exp Bot.

[CR39] Van Baarlen P, Verduijn M, Van Dijk PJ (1999). What can we learn from natural apomicts?. Trends Plant Sci.

[CR40] Wędzony M (1996). Fluorescence microscopy for botanists (in Polish).

[CR41] Wróbel-Marek J, Kurczyńska E, Płachno BJ, Kozieradzka-Kiszkurno M (2017) Distribution of symplasmic transport fluorochromes within the embryo and seed of *Sedum acre* L. (Crassulaceae). Planta 245(3):491–505 doi:10.1007/s00425-016-2619-y 10.1007/s00425-016-2619-y PMC5310571 27888360

