# Supplementary figures and images for: Integument cell gelatinisation—the fate of the integumentary cells in Hieracium and Pilosella (Asteraceae)
Source: Protoplasma. 2017 May 15;254(6):2287–94. doi: 10.1007/s00709-017-1120-1 (PMC5653734; doi:10.1007/s00709-017-1120-1)

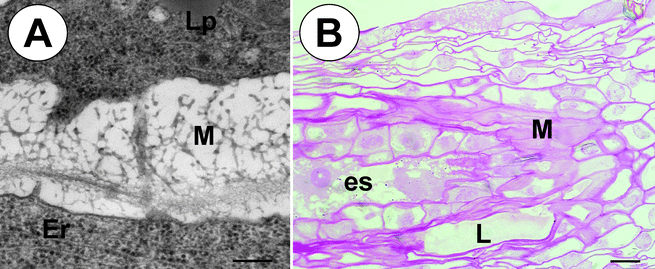

Supplement: Supplementary file 1 — A. Hieracium alpinum, TEM. Ultrastructure of the mucilage cells: lipid droplet (Lp), mucilage (M), endoplasmic reticulum (Er); bar = 200 nm. B Pilosella officinarum, Light microscopy. Ovule after PAS reaction, note PAS-positive material in mucilage cells (M); embryo sac (es), mucilage cavities (L); bar = 10 μm. (GIF 142 kb) [file 709_2017_1120_Fig8_ESM.gif]

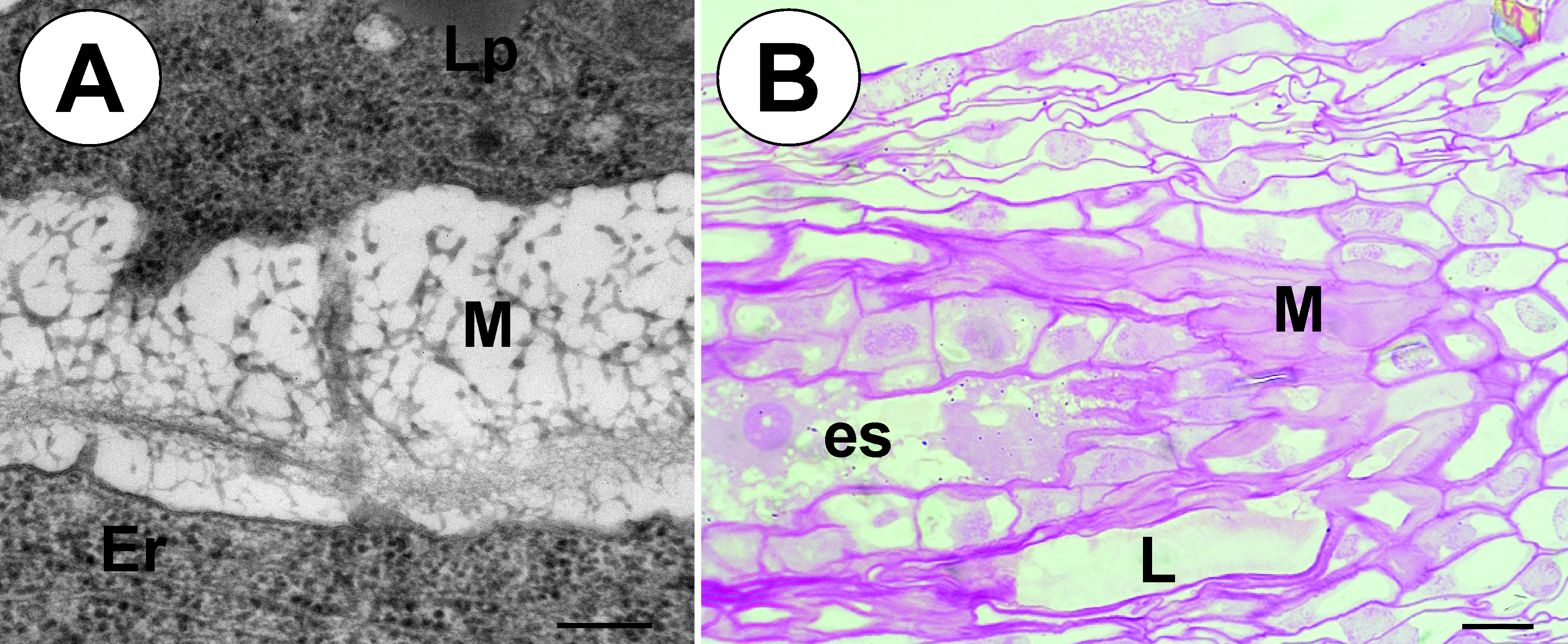

Supplement: Supplementary file 2 — High resolution image (TIFF 17239 kb) [file 709_2017_1120_MOESM1_ESM.tif]
